# Enrichment-Free Single-Cell Detection and Morphogenomic Profiling of Myeloma Patient Samples to Delineate Circulating Rare Plasma Cell Clones

**DOI:** 10.3390/curroncol29050242

**Published:** 2022-04-21

**Authors:** Libere J. Ndacayisaba, Kate E. Rappard, Stephanie N. Shishido, Carmen Ruiz Velasco, Nicholas Matsumoto, Rafael Navarez, Guilin Tang, Pei Lin, Sonia M. Setayesh, Amin Naghdloo, Ching-Ju Hsu, Carlisle Maney, David Symer, Kelly Bethel, Kevin Kelly, Akil Merchant, Robert Orlowski, James Hicks, Jeremy Mason, Elisabeth E. Manasanch, Peter Kuhn

**Affiliations:** 1 Convergent Science Institute in Cancer, Michelson Center for Convergent Bioscience, University of Southern California, Los Angeles, CA 90089, USA; ndacayis@usc.edu (L.J.N.); rappard@usc.edu (K.E.R.); sshishid@usc.edu (S.N.S.); ruizvela@usc.edu (C.R.V.); matsumon@usc.edu (N.M.); rnevarez@usc.edu (R.N.); msetayes@usc.edu (S.M.S.); naghdloo@usc.edu (A.N.); ching-ju.hsu@bcm.edu (C.-J.H.); cmaney@usc.edu (C.M.); jameshic@usc.edu (J.H.); masonj@usc.edu (J.M.); 2 Keck School of Medicine, University of Southern California, Los Angeles, CA 90033, USA; kevin.kelly@med.usc.edu; 3 Department of Hematopathology, University of Texas MD Anderson Cancer Center, Houston, TX 77030, USA; gtang@mdanderson.org (G.T.); peilin@mdanderson.org (P.L.); 4 Department of Lymphoma/Myeloma, Division of Cancer Medicine, University of Texas MD Anderson Cancer Center, Houston, TX 77030, USA; desymer@mdanderson.org (D.S.); rorlowski@mdanderson.org (R.O.); eemanasanch@mdanderson.org (E.E.M.); 5 Department of Pathology, Scripps Clinic Medical Group, La Jolla, CA 92037, USA; bethel.kelly@scrippshealth.org; 6 Department of Medicine, Cedars Sinai Medical Center, Los Angeles, CA 90048, USA; akil.merchant@cshs.org; 7 Institute of Urology, Catherine & Joseph Aresty Department of Urology, Keck School of Medicine, University of Southern California, Los Angeles, CA 90033, USA; 8 Norris Comprehensive Cancer Center, Keck School of Medicine, University of Southern California, Los Angeles, CA 90033, USA; 9 Department of Biomedical Engineering, Viterbi School of Engineering, University of Southern California, Los Angeles, CA 90089, USA; 10 Department of Aerospace and Mechanical Engineering, Viterbi School of Engineering, University of Southern California, Los Angeles, CA 90089, USA; 11 Department of Biological Sciences, Dornsife College of Letters, Arts, and Sciences, University of Southern California, Los Angeles, CA 90089, USA

**Keywords:** multiple myeloma, circulating plasma cells, morphogenomics, rare single cell, liquid biopsy, HDSCA, peripheral blood, bone marrow aspirate, multimodal data

## Abstract

Multiple myeloma is an incurable malignancy that initiates from a bone marrow resident clonal plasma cell and acquires successive mutational changes and genomic alterations, eventually resulting in tumor burden accumulation and end-organ damage. It has been recently recognized that myeloma secondary genomic events result in extensive sub-clonal heterogeneity both in localized bone marrow areas and circulating peripheral blood plasma cells. Rare genomic subclones, including myeloma initiating cells, could be the drivers of disease progression and recurrence. Additionally, evaluation of rare myeloma cells in blood for disease monitoring has numerous advantages over invasive bone marrow biopsies. To this end, an unbiased method for detecting rare cells and delineating their genomic makeup enables disease detection and monitoring in conditions with low abundant cancer cells. In this study, we applied an enrichment-free four-plex (CD138, CD56, CD45, DAPI) immunofluorescence assay and single-cell DNA sequencing for morphogenomic characterization of plasma cells to detect and delineate common and rare plasma cells and discriminate between normal and malignant plasma cells in paired blood and bone marrow aspirates from five patients with newly diagnosed myeloma (*N* = 4) and monoclonal gammopathy of undetermined significance (n = 1). Morphological analysis confirms CD138+CD56+ cells in the peripheral blood carry genomic alterations that are clonally identical to those in the bone marrow. A subset of altered CD138+CD56- cells are also found in the peripheral blood consistent with the known variability in CD56 expression as a marker of plasma cell malignancy. Bone marrow tumor clinical cytogenetics is highly correlated with the single-cell copy number alterations of the liquid biopsy rare cells. A subset of rare cells harbors genetic alterations not detected by standard clinical diagnostic methods of random localized bone marrow biopsies. This enrichment-free morphogenomic approach detects and characterizes rare cell populations derived from the liquid biopsies that are consistent with clinical diagnosis and have the potential to extend our understanding of subclonality at the single-cell level in this disease. Assay validation in larger patient cohorts has the potential to offer liquid biopsy for disease monitoring with similar or improved disease detection as traditional blind bone marrow biopsies.

## 1. Introduction

Multiple myeloma (MM) is a plasma cell (PC) neoplasm that is the second leading hematologic malignancy worldwide, accounting for 34,920 new cases and 12,410 deaths annually in the US alone [1]. Nearly all MM cases are preceded by monoclonal gammopathy of undetermined significance (MGUS) [2]. MM initiates when germinal center B-cells undergoing normal affinity maturation encounter an error either in somatic hypermutation of the immunoglobulin heavy chain (IGH) locus, or class-switch recombination [3]. These distinct initial genetic errors give rise, respectively, to a set of characteristic and mutually exclusive chromosome 14-based translocations involving the IGH locus (14q32), primarily t(11;14), t(4;14), t(14;16) [4], and characteristic hyperdiploidy of odd-numbered chromosomes. Together, these large chromosomal structural events define two genomic categories that comprise the hallmarks of myeloma genetics [3,5]. Over time, monoclonal PCs acquire secondary mutational changes and evade the immune system to form multiple focal bony lesions which can eventually spread beyond the bony medullary space into soft tissues and organs [5,6,7,8]. Despite advances in therapeutic modalities, nearly all MM cases relapse, and the disease remains mostly incurable in large part due to the emergence of resistant genomic clones both as part of the genomic heterogeneity within lesions [9,10], and anatomically spatial variability between lesions [11]. Sampling and analysis of common/hallmark myeloma cytogenetic events from bone marrow PCs is key to MM clinical diagnosis, staging, and disease monitoring [12,13,14]. MM diagnosis and stratification are accomplished by genomic profiling of CD138+ cells isolated from bone marrow aspirates (BMAs) by clinical karyotyping and Fluorescent In Situ Hybridization (FISH) assays to identify the presence of canonical trisomies in odd-numbered chromosomes (hyperdiploid disease) and for translocations (non-hyperdiploid disease) which together allows for patient stratification and therapy selection, particularly for high-risk disease [13,15].

As aberrant myeloma cells evolve, they are able to relocate from their bone marrow niche into the peripheral blood circulation which offers a unique opportunity to evaluate the myeloma tumor in a non-invasive manner. In this respect, a robust unbiased method for detection and morphogenomic description and quantification of MM circulating tumor cells (MM CTCs) of interest would be enabling particularly in discriminating abnormal from normal PCs. While peripheral blood (PB) is routinely sampled to measure the myeloma monoclonal (M) protein and other soluble molecules as part of the standard diagnostic workup, genomic profiling of MM CTCs is not yet routinely used in clinical care. Recent works characterizing MM CTCs have focused on the surface marker CD138 (syndecan-1) for identification and isolation and have provided significant insights into the nature and role of these cells in myeloma. MM CTCs have been found to correlate with disease progression and survival and provide a new avenue for disease risk stratification in precursor myeloma and disease monitoring (minimal residual disease [MRD]) for relapsed/refractory (RRMM) patients [16,17,18,19,20]. Current bulk and single-cell technologies to detect and isolate CD138+ MM CTCs include flow cytometry (FC) [17,21], the combined CellSearch-DEPArray method (Silicon Biosystems) [22,23], and custom microfluidics [24].

While multiplex flow cytometry enables quantitative separation of cell types by marker-based gating and further characterization of disease, an enrichment step is required to find and isolate single cells of interest, thus limiting its application in contexts where target cells are in low abundance where unbiased slide-based methods are optimal for single-cell profiling. Similarly, the CellSearch-DEPArray method also relies on initial PC enrichment by CD138 and CD38 positivity and may likewise lack the sensitivity and accuracy needed for some MGUS and RRMM patient populations. Furthermore, the high degree of heterogeneity in marker expression by PCs requires an unbiased approach capable of detecting diverse PC populations based on their morphological characteristics. To resolve the challenges in enrichment-based methods for PC detection, Zhang et al. developed an enrichment-free single-cell detection assay to characterize MM CTCs in PB draws of newly diagnosed (NDMM) patients using CD138, CD45, and pS6 (ribosomal protein S6) on the Epic Sciences Platform [25], the commercial version of the High-Definition Single Cell Assay (HDSCA) workflow, an identification method originally developed for CTC detection and characterization in epithelial cancers [26,27,28,29,30,31]. The Epic Sciences team applied the “no-cell-left behind” approach and performed extensive validation, linearity, qualification, and reproducibility experiments for an HDSCA-based MM CTC assay to detect and characterize circulating rare cells in myeloma. While the morphological analysis and enumeration of HDSCA MM CTCs provided insights into the morphological phenotypes and distribution of CD138 and pS6 expressing rare cells in NDMM PB, genomic profiling to validate the technology’s capability to distinguish normal and abnormal PCs has not been performed. Furthermore, morphogenomic validation to evaluate clonal and subclonal variability and to correlate single copy number alteration profiles to clinical diagnostic and disease monitoring remains to be performed in this enrichment-free methodology.

In this study, we modified the Epic Science’s MM CTC methodology to include CD56 rather than pS6 in a 4-plex (CD138, CD56, CD45, and DAPI) immunofluorescence assay adapted to the 3rd generation HDSCA (Chai et al.) to detect, sequence, and describe the morphogenomic profiles of PCs in NDMM and MGUS PB and BMA samples. We define MM CTC and bone marrow PC (BMPC) candidates as CD138+ nucleated cells, with the expression of CD56 further subtyping normal and abnormal PCs. PC malignancy was validated via next-generation single-cell sequencing, in which single-cell copy number variation (scCNV) chromosomal events were correlated to results from clinical FISH and cytogenetic analysis toward clinical validation. Consistent with prior work, we detected candidate MM CTCs and BMPCs that possess the canonical morphological immunophenotype, namely CD138+ cells that are predominantly larger than other white blood cells (WBCs) and present eccentric nuclei. scCNV analysis validated that candidate MM CTCs harbor chromosomal alterations detected with standard clinical diagnostic cytogenetic methodologies and with additional single-cell subclones with genomic profiles not detected by clinical methods. The reported data provide additional technical validation of an unbiased single-cell liquid biopsy approach for the detection and genomic characterization of MM CTCs with promising applications in early disease (MGUS) and in other myeloma patient subgroups where clonal malignant cells can be in low abundance.

## 2. Materials and Methods

### 2.1. Patient Enrollment and Sample Acquisition

**Figure 1:**
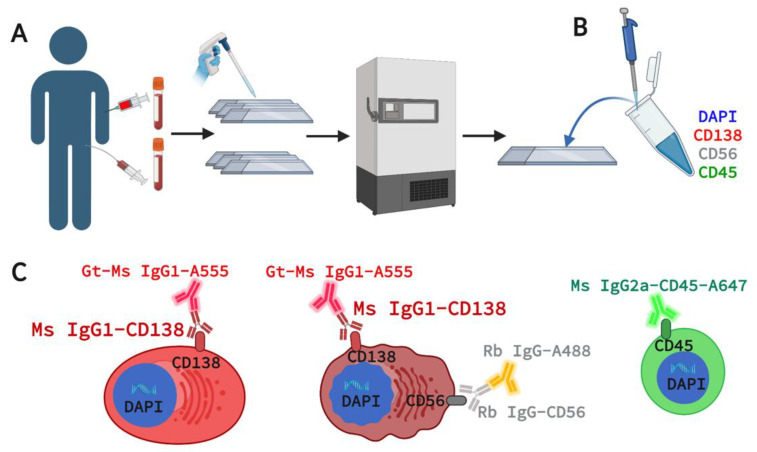
HDSCA-based 4-plex Immunofluorescence for Rare Single Cell Detection in Plasma Cells. (**A**) Paired sample acquisition, processing, and cryo-banking. (**B**) Slides are stained with a cocktail of antibody markers targeting cells of interest. (**C**) Immuno-targeting for normal PC (CD138+ only), candidate malignant PCs (CD138+CD56+), and common WBCs (CD45+ only). Gt = Goat, Ms = Mouse, Rb = Rabbit, Ig = immunoglobulin, DAPI = 4′,6-diamidino-2-phenylindole, A555 = Alexa Fluor^®^ 555, A488 = Alexa FluorPLUS^®^ 488, A647 = Alexa647.

All patients enrolled in this study provided informed consent following an Institutional Review Board (IRB)-approved protocol (PA18-1073) and were accrued at the University of Texas MD Anderson Cancer Center. Five patients (four NDMM and one MGUS) enrolled prospectively between 5 April 2019 and 29 May 2019 provided paired blood and bone marrow samples. To obtain an accurate diagnosis, all patients underwent a bone marrow biopsy, blood work, 24-hour urine collection, and advanced whole-body imaging. The same samples from each patient were analyzed via FC by MD Anderson Cancer Center as part of the standard MM diagnostic workup. Four normal blood donor (NBD) samples from individuals with no previously known pathologies were procured from the Scripps Clinic Normal Blood Donor Service in La Jolla, CA. BMA and PB specimens were drawn and collected using anti-coagulated preservative tubes (Cell-Free DNA blood collection tube, Streck) and shipped to the USC Michelson Convergent Science Institute in Cancer (CSI-Cancer) via FedEx overnight and processed as previously described using the established and validated HDSCA workflow [26,30,31,32]. In short, samples underwent red blood cell lysis using an isotonic ammonium chloride buffer and all nucleated cells were plated as a monolayer onto custom-made glass slides (Marienfeld, Germany) at approximately 3 million cells per slide prior to cryopreservation (Figure 1A). Slides were retrospectively thawed for immunofluorescence staining (Figure 1B).

### 2.2. Marker Selection for 4-Plex Immunofluorescence Assay

The goal of this assay was to detect PCs using well-established myeloma and immune markers with CD56 expression used for further discrimination of normal from malignant cells. The 4-plex staining panel consists of CD138, CD56, CD45, and DAPI (4′,6-diamidino-2-phenylindole) for targeting cells of interest (Figure 1C). For PC detection in PB and BMA samples, CD138 was selected for its unique expression on PCs within the immune system [32] and its role in MM disease progression [33]. CD56 (Neural Cell Adhesion Marker 1, N-CAM-1) is a well-established MM biomarker expressed by malignant PCs in over 70% of MM patients as studied by FC [34,35]. CD45 was chosen as a WBC marker and MM PC exclusionary marker, as over 80% of patients have CD45- MM BMPCs [36]. DAPI is used to identify all nucleated cells, including PCs and surrounding common WBCs. While pS6 (used in Zhang et al.) drives PI3K/AKT activation in malignant PCs, this pathway-driven process may also be observed in other cells depending on their activation states. As such, it is not used in clinical FC assays as surface markers are prioritized for immunophenotyping of normal and malignant PCs for clinical utility [20,37,38,39] and we chose CD56 as the substitute for pS6 in this assay. For this assay, we procured mouse anti-human IgG1 CD138 (Exbio, clone A-38, Cat #, cat# 10-520-C100, Czech Republic), rabbit anti-human IgG CD56 (Invitrogen, cat # 701379), and directly conjugated mouse anti-human CD45-Alexa647 (AbD Serotec, act# MCA87A647, Raleigh, NC, USA) along with mouse anti-human Alexa Fluor^®^ 555 (Invitrogen, Waltham, MA, USA) and goat anti-rabbit Alexa FluorPLUS^®^ 488 (Invitrogen, Waltham, MA, USA) secondary antibodies targeting CD138 and CD56 respectively.

### 2.3. Assay Staining and Validation in Cell Lines and Spiked NBD Samples

Immunoglobulin E lambda myeloma-derived U266 cell line (gift from Dr. Akil Merchant; U266B1 TIB-196TM), immunoglobulin A lambda myeloma-derived MM.1S cell line (ATCC^®^ CRL-2974™), and T-lymphocyte-derived Jurkat cell line (ATCC^®^ TIB-152™) were cultured according to manufacturer’s specification. Slides for assay development were generated with pure cell lines (U266, MM.1S, and Jurkat) to test the expression of CD138 in myeloma and control cell lines.

To compare the expression of CD138 in cell lines compared to normal WBCs, U266 cell line was spiked into NBD blood at a 1:100 dilution. To establish the optimal concentration for anti-CD138, anti-CD56, and anti-CD45 antibodies, we performed antibody titrations from concentrations of 0 μg/mL to 10 μg/mL using contrived samples with the U266, MM.1S, and Jurkat cell lines. For specificity of the anti-CD138 antibody, we used Jurkat cells as a CD138- control. For specificity of the mouse anti-human Alexa Fluor^®^ 555 secondary antibody, we used a no primary antibody control in which the anti-CD138 antibody is omitted from staining. Additional experiments for assay sensitivity and reproducibility were previously performed and demonstrated by Zhang et al., with an accuracy of 97.5% and the capability of detecting one spiked CD138+ cell in 3 million cells [25].

### 2.4. Assay Staining and Validation in Patient PB and BMA

Provided the final reagents and conditions above, matched PB and BMA slides were assayed for MM CTCs and BMPCs in patient samples. Following previously established protocols [26], slides were thawed after −80 °C storage, fixed using 2% paraformaldehyde for 20 min, and washed using TBS buffer. The slides were incubated with 10% goat serum for 30 min to block non-specific binding sites for secondary antibodies. Next, the slides were incubated with the primary antibody mix consisting of 2 µg/mL of CD138, 5 µg/mL of rabbit anti-human CD56, and 1.6 µg/mL of mouse anti-human CD45-Alexa647 for 1 h at room temperature. Following TBS washes, secondary antibodies goat anti-mouse Alexa Fluor^®^ 555 (1:500) and goat anti-rabbit Alexa FluorPLUS^®^ 488 (1:500) were added for CD138 and CD56 staining, respectively, with DAPI for 40 min at room temperature. As previously described [26,30,31,40], the slides were mounted with live-cell media, cover slipped, and sealed for downstream microscopy imaging and technical analysis.

### 2.5. Imaging and Technical Analysis for Rare Cell Detection and Cell Classification

Each slide was imaged at 100× magnification using a custom-made fluorescent scanning microscope, generating 2304 frames per slide with a total of approximately 9200 images across four fluorescent channels [26]. The image data set was analyzed via a custom rare event detection algorithm known as OCULAR (Outlier Clustering Unsupervised Learning Automated Report) [41]. OCULAR utilizes the principles of image processing for single-cell segmentation and feature extraction, dimensionality reduction, and unsupervised clustering approaches to distill morphologically distinct rare cells from common cells. Briefly, 761 features per single cell are extracted using EBImage [42]. K-Nearest Neighbor is applied to classify cells based on marker signal intensity, cell shape, and geometry and subsequently used to filter all distinct cell populations (i.e., MM CTCs and BMPC candidates) from surrounding common cells (CD138-CD45+ WBCs). For final classification and enumeration, a trained technician confirmed the automated classification by visually inspecting the expression of each marker and the morphological integrity of the cell and assigning it the correct candidate morphological subtype. All cells manually classified were re-confirmed by two additional individuals for cross-validation and reproducibility. MM CTC and BMPC candidates are defined by eight subtypes, as expanded from [25]:

- CD138+
- CD138+CD56+
- CD138+CD45+
- CD138+CD56+CD45+
- CD138−: any cells larger than surrounding WBCs and have eccentric nuclei
- Apoptotic: any CD138+ cells with condensed DAPI pattern and/or blebbing as previously described [25,26]
- PC clusters: cluster consisting of two or more CD138+ cells
- Binucleated PC: CD138+ cells presenting two morphological distinguishable nuclei

Additional rare cells of interest not categorized as MM CTCs or BMPCs were tracked, characterized, and enumerated using the OCULAR software previously described (Chai et al. 2021) and were not included in this study. Each cell category is reported as total count of cells per mL of blood.

### 2.6. Single-Cell Sequencing and CNV Analysis

To generate single-cell genomic data for morphogenomic characterization, candidate CD138+ cells (and morphological subsets thereof with additional representative cells from groups of interest) were isolated and sequenced using protocols previously developed and described by our laboratory [28,43,44]. In short, candidate single cells are isolated using a robotic micromanipulator system and subjected to whole-genome amplification (WGA; Sigma Aldrich, St. Louis, MI, USA) and library construction as described [28,43,44]. Amplified DNA was purified using the QIAquick PCR purification kit (Qiagen, Hilden, Germany) and resulting DNA was quantified using the Quibit Fluorometer (Thermo Fisher). Indexed Illumina sequencing libraries were constructed and barcoded using the NEBNext Ultra DNA Library Preparation Kit (New England Biolabs, Ipswich, MA, USA). The amplified DNA fragments with target size were sequenced at either the USC Dornsife Sequencing Core or Fulgent Genomics (Temple City, CA, USA) to generate approximately 500,000 mapped reads. Sequenced reads were analyzed using our previously described CNV pipeline for reference genome mapping, read alignment, and single-cell ploidy determination [40,41,45]. For each sequenced and mapped cell, 2 copy numbers were normal and any cell whose profile deviate was considered altered. Chromosomal alterations were evaluated across the cells to establish the clonal relationship between cells from the same slide.

### 2.7. Karyotyping and Fluorescent in Situ Hybridization (FISH) for Clinical Diagnosis

Standard FISH was performed for all patients as part of the diagnostic workup for monoclonal gammopathies. Bone marrow aspirates were collected at study enrollment and CD138+ cells were enriched using RoboSep-STM (Stem Cell Technologies, Vancouver, BC, Canada) plasma cell enrichment. This increases sensitivity for detecting cytogenetic abnormalities associated with plasma cell neoplasms. For successful FISH analysis for detecting myeloma-associated abnormalities, the clinical laboratory requires the bone marrow to contain neoplastic plasma cells detected by flow cytometry greater than 0.05% of the total cells analyzed, and greater than 25% of plasma cells are aberrant neoplastic plasma cells. The tests were developed, and their performance characteristics were determined by M.D. Anderson Cancer Center Cytogenetics Laboratory as required by the CLIA’88 regulations. Chromosomal analysis (karyotyping) was performed on metaphase cells prepared from bone marrow (BM) aspirate specimens cultured for 24 h without mitogens or for 72 h with lipopolysaccharide (LPS), using standard techniques. Twenty Giemsa-banded metaphases were analyzed, and the results were reported using the International System for Human Cytogenetic Nomenclature (ISCN 2020). FISH for common abnormalities associated with plasma cell myeloma was performed on interphase nuclei obtained from cultured BM cells using dual-color FISH probe sets designed to detect the rearrangements of t(4;14)/IGH::FGFR3, t(11;14)/IGH::CCND1, and copy number changes of CDKN2C/CKS1B, RB1/13q34; and TP53/CEP17, according to the manufacturer’s instructions (Abbott Molecular, Abbott Park, IL, USA). Two hundred nuclei are analyzed for each probe set, and the results were reported based on the cut-off values established by our clinical cytogenetics laboratory.

### 2.8. Correlating scCNV to Clinical Cytogenetics

To map scCNV chromosomal alterations detected by our 4-plex assay to the results from the clinical diagnostic workup, we focused on the 12 common cytogenetic alteration events probed in the MD Anderson Cancer Center targeted clinical FISH workup protocol. For every single cell, we assessed the presence or absence of each cytogenetic event and quantified how many cells harbor genomic alterations observed using standard clinical methods.

### 2.9. Statistical Analysis

All data analysis was performed in the R programming language (R version 3.6.3). The intersection analysis in rare cell count and cytogenetic-based validation was performed using the ComplexUpSetR package. Comparison of total MM CTC count between NDMM and NBD samples was computed using the Wilcoxon signed-rank test with a 95% confidence interval.

## 3. Results

### 3.1. Expression of CD138 and CD56 in U266, MM.1S, and Jurkat Cells Spiked in Normal Blood

From antibody validation experiments on U266, MM.1S, and Jurkat (negative control) cells, the final optimal concentration for anti-CD138 was found to be 2 µg/mL and 5 µg/mL for anti-CD56. In comparative stains, Jurkat cells show no expression of CD138, while both MM.1S and U266 are positive for CD138 (Figure S1A) with a statistically significant difference in CD138 intensity (Figure S1B,C). Omission of the primary antibody (0 µg/mL of CD138) yielded no CD138+ cells in both positive and negative cell lines in titration experiments. In NBD spike-in experiments in U266, CD138+CD56-CD45− and double-positive CD138+CD56+ U266 cells were detected with morphology that is distinct from surrounding CD138−CD56−CD45+ common WBCs (Figure S1D). Consistent with the histological description of MM PCs, the U266 cells were CD138+CD56+CD45- and larger than surrounding WBCs with the canonical eccentric nucleus (Figure S1D). A subset of CD138+CD45+ U266 cells was also detected consistent with cell line marker expression variability. Together, these data confirm the specificity of the antibodies consistent with and supplementing sensitivity, reproducibility, specificity, and analytical validation work for HDSCA technology, as previously reported [25,41].

### 3.2. Patients and Study Cohort

The patient cohort is comprised of one male MGUS (age 78), one female NDMM (age 63), and three male NDMM (ages 80, 54, and 66) with NBD controls consisting of two males (ages 48 and 65) and two females (ages 54 and 59). By clinical BMA histopathology for NDMM and MGUS, all BMPCs are CD138+ and, with the exception of patient MM03, also CD56+. MM01 and MGUS presented CD45+ histopathology, and both cases have the lowest percentages of BMPCs. Detailed characteristics of patients in this study are shown in Table 1.

### 3.3. Morphological Characterization, Classification, and Enumeration of MM CTCs and BMPCs

**Figure 2:**
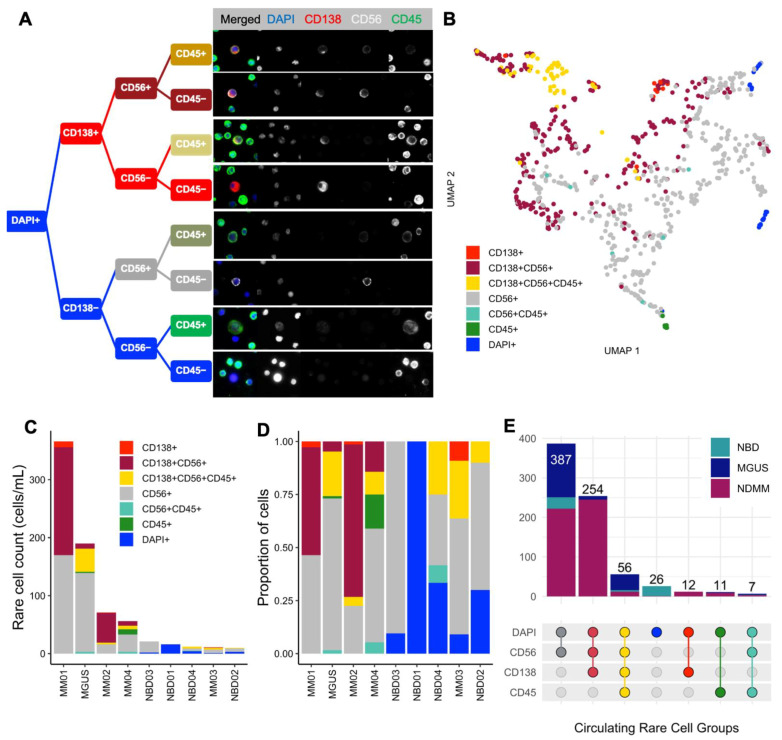
Rare Single-Cell Classification and Enumeration. (**A**). Decision tree structure classification of DAPI+ cells showing all candidate cell groups detected, with respect to immunofluorescence expression, along with representative images (400× magnification). (**B**) UMAP projection of all detected circulating rare cells colored by their respective classification groups. Using the 761 features extracted with EBImage, Uniform Manifold Approximation Manifold (UMAP) [47,48], a non-linear dimensionality reduction method, was used to represent circulating rare cells and their corresponding classification groups in two-dimensional space for analysis. (**C**) Enumeration and (**D**) proportional distribution of circulating rare cells (cells/mL) for each cell group across all samples. (**E**) Distribution of circulating rare cell counts across NDMM (*N* = 4), MGUS (*N* = 1), and NBD (*N* = 4) grouped by rare event group based on marker expression. Color scheme for cell classification groups is consistently preserved.

As an unbiased, enrichment-free immunofluorescence assay supported by a previously published rare cell detection computational algorithm [41], the plasma cell assay detects a heterogeneous mixture of morphologically distinct cells from the BMA and PB samples. Beyond CD138+ cells, additional rare cells are identified by a combination of cellular and physical morphology and their CD56 and CD45 expression. A decision tree representation (Figure 2A) and UMAP projection (Figure 2B) separating distinct cells based on their geodesic distance in the Riemannian manifold presents cell groups of all the circulating rare cells detected in PB of all the samples (MGUS, NDMM, and NBD) in this study. The CD138+ rare cell groups closely clustered separately from the CD56+ and CD45+ rare cells.

Quantitative enumeration of detected circulating rare cells found that all 258 CD138+CD56+CD45− candidate MM CTCs are exclusively found in NDMM and MGUS patients, with MM01 accounting for 186 cells, followed by MM02 with 51 cells; MM03 does not have any such double-positive cells (Figure 2C–E). The CD138−CD56+CD45− cells seen across samples including NBD are likely characteristics of normal CD56+ NK T cells. Intersection analysis (Figure 2E) shows the distribution of detected cells for each channel and combination thereof when compared between individual patients and across disease states versus NBD. CD138+CD56−CD45− are exclusively found in myeloma patients. While CD138+CD56+CD45+ cells are predominantly in myeloma patients, they are also found in NBD (Figure 2E), possibly being normal PCs that are not fully differentiated. Since MM bone marrow is not a rare event space, the analysis focused on cell characterization instead of enumeration.

**Figure 3:**
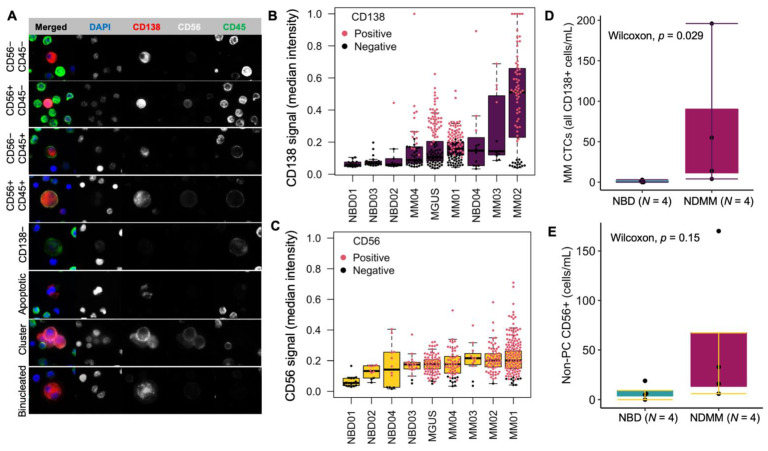
Morphological Characterization of MM CTCs and BMPCs. (**A**) Microscopy images of representative cells from morphological subtypes of PB and BMA cells. (**B**) CD138 and (**C**) CD56: signal intensity in circulating rare cells across samples, colored by positive or negative marker expression based on manual classification. (**D**) Statistical comparison of MM CTC count between NDMM and NBD. (**E**) Statistical comparison of non-plasma cell CD56 positive cells (sum of CD56+ and CD56+CD45+ cells).

Focusing on CD138+ cells to further characterize MM CTCs and BMPCs, the immunophenotypes and morphology of MM CTCs (CD138+ circulating rare cells) and BMPCs include subsets with CD56 and CD45 signals with BMPCs showing PC clusters, binucleated PCs, apoptotic PCs, and CD138− candidate PCs, as previously observed [25] (Figure 3A). Looking at the CD138 signal in circulating rare cells of different patients, the median intensity across rare cells was compared between patients. The intensity of CD138 and CD56 is consistently higher in cells from patient samples as compared to NBDs, confirming the specificity of the assay for rare PC detection in PB of patients (Figure 3B, C). Manual classification for CD138 and CD56 (red = positive, black = negative) is also consistent with a signal expression showing that, except for NBD04, which had a PC candidate with a CD138 signal significantly above the maximum intensity, patient blood samples constitute the only specimens with more than three cells that the technical analysis found to be positive (Figure 3B).

While the quantification of circulating CD138+ cells in the blood of NBDs has not been extensively investigated and well established, two studies have reported 0–5 CD138+ cells/mL in circulation [36,49,50]. Among the four controls in this study, NBD04 had three CD138+ cells and NBD02 had one, with zero cells detected in the other two donors (Figure 2E). All patient samples exhibited CD138+ cells (MM CTCs) with a minimum of four cells/mL in MM03 and a high count of 196 cells in MM01. Accordingly, the total number of MM CTC candidate cells is significantly higher in NDMM compared to NBD samples (*p* = 0.029) (Figure 3D). Despite higher counts in NDMM compared to NBD for circulating non-PC CD56+ (candidate NK T cells), the difference in total cell count between the two pathologies is not statistically significant (*p* = 0.15) (Figure 3E). Notably, the CD138+CD56+CD45− cells, which constitute the candidate circulating malignant PCs, were found exclusively in NDMM and MGUS samples, supporting that the detected MM CTCs are the primary candidate malignant PCs and further confirming assay specificity.

### 3.4. scCNV for Morphogenomic Validation of Malignant Phenotypes in Detected MM CTCs and BMPCs

**Figure 4:**
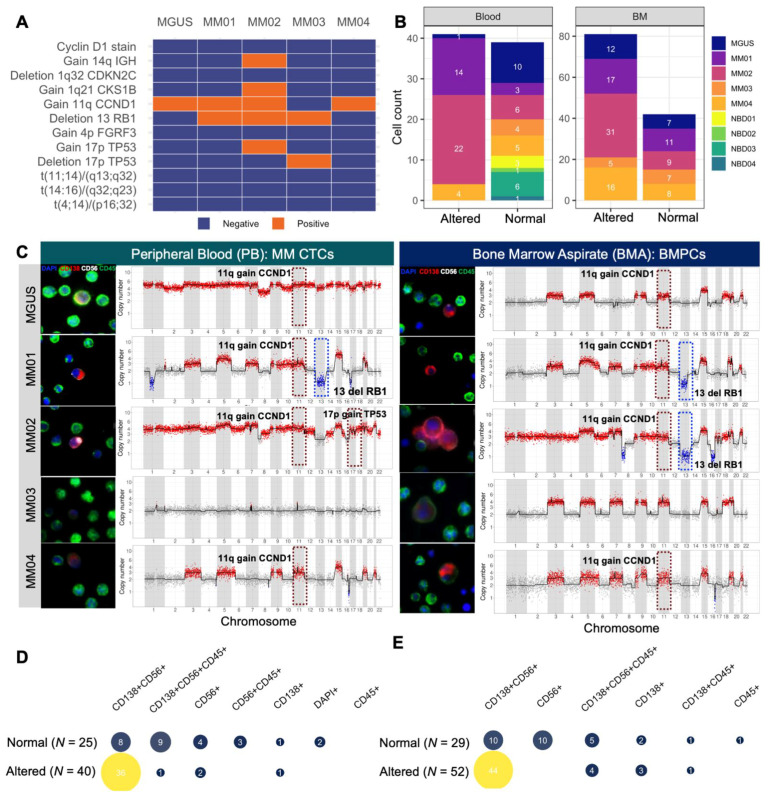
Morphogenomic validation of detected candidate MM CTCs and BMPCs. (**A**). Clinical observations of the status of 12 common cytogenetic events detected by FISH karyotyping across patients’ bone marrow samples. (**B**) Distribution of scCNV profiles across patients and sample types. (**C**) Representative morphological phenotype with corresponding scCNV profiles of subclones containing clinically determined key cytogenetic events. The scCNV profile is for the single CD138+ cell in the corresponding IF image. The red and blue hashed rectangles indicate an alteration event where the patient is also positive by clinical cytogenetics detection. There were no altered cells in MM03 blood. (**D**,**E**). Single-cell count between normal and altered genomic profiles across morphological groups. A cell is considered altered if the CNV profile contains at least one discernable chromosomal aberration or ploidy.

To validate that the candidate malignant PCs harbor genomic aberrations, single-cell next-generation sequencing analysis was performed on target cells of interest across morphological groups. In total, from paired PB and BMA samples of the four NDMM patients, 165 cells were isolated and successfully underwent WGA, sequencing, and analysis, with an additional 30 cells from paired PB and BMA samples of the one MGUS case. For the NBD controls, a total of 12 cells from four donors were sequenced and analyzed. For controls within a sample, 1 to 2 WBCs (CD45+ only) from each patient sample are also included in the pool of single cells selected for sequencing. Furthermore, data from clinical diagnostic FISH cytogenetic results (Figure 4A and Table 1) were mapped to single-cell CNV profiles of altered cells. The sequenced cell counts across study subjects and samples with the corresponding scCNV profile status are shown in (Figure 4B). Representative CD138+ cells and their respective scCNV profiles (Figure 4C) from PB (left) and BMA (right) validate that the detected candidate MM CTCs and MMPCs (CD138+CD45− cells) are malignant PCs. Representative positive cytogenetic events from the clinical FISH analysis of the BM plasma cells (Figure 4A) are marked with a hashed rectangle (blue for deletion and red for gain). To join morphological observations with CNV results, quantification of normal and altered cells across morphological types is presented in (Figure 4D for PB, Figure 4E for BM). Except for MM03 PB, all of the BMA and PB samples for the four NDMM patients and the MGUS case have CD138+ cells with genomic alterations consistent with the characteristic myeloma hyperploid karyotypes exhibiting copy number alterations among the odd-numbered chromosomes [14,51].

Consistent with the malignant PC phenotype, in both sample types, all altered cells are CD138+, except for three CD138-CD56+ cells in PB. Additionally, the CD138+CD56+CD45− is the predominant morphotype with the most genomically altered cells with 36/44 (81.8%) of cells in PB and 44/54 (81.4%) cells in the BMA being altered (Figure 4D,E). As expected in NBD controls, all sequenced CD138+ cells from the NBDs have normal CNV profiles, confirming that they are normal PCs in circulation. Furthermore, at least one WBC (CD45+ only) per sample was sequenced and presented no alterations. Figure S2 contains the detailed chromosomal scCNV profiles of all sequenced cells across the study cohort. On a patient-by-patient basis, clonally altered cells were found in MM01 PB (14/17 cells sequenced; 82.3%) and BMA (17/28 cells sequenced; 60.7%). The same five subclones could be discerned in both PB and BMA, with the dominant subclone exhibiting a canonical hyperdiploid trisomy profile with gains in chromosomes 3, 5, 7, 9, 11, 15, 19, and 10, and with other subclones containing additional losses in 1p and 13q. In MM02, 22/28 (78.6%) PB cells and 32/40 (80%) BMA cells were altered with five subclones in both samples. The dominant subclone harbors copy number gains in chromosomes 5, 7, 15, and 21 and loss of chromosomes 8, 12, 13, 14, 16, 20, and 22. All 22 altered cells in MM02 PB are polyploid, with 19/22 (86.4%) cells being triploid, 2/22 (9.1%) cells tetraploid, and 1/22 (4.5%) cells pentaploid. Of the 32 altered cells in the BM, 3/32 (9.4%) are diploid, 26/32 (81.2%) are triploid, and 3/32 (9.4%) are tetraploid. In MM03, all four sequenced PB cells have a normal CNV profile, and 5/12 (41.7%) BMA cells are altered with a unique clonal profile for the altered cells carrying gains in 3, 5, 7, 9, 11, 15, 18, and 19. In MM04, 4/9 (44.4%) PB and 16/27 (59.2%) BMA cells showed clonal alterations. The dominant subclone was found in both specimens and, consistent with trisomic hyperdiploidy, harbor gains in 3, 5, 7, 9, 11, 15, 19, and 21 and 16q loss with a secondary subclone that carries losses in 1p and 4q. In the MGUS PB, CNV analysis revealed one (1/11 cells sequenced, 9.0%) circulating aberrant MM CTC with a pentaploid profile and a loss of chromosome 8 while 12/19 (63.1%) malignant BMPCs exhibited a CNV profile with the classic gains of odd-numbered chromosomes; 3, 5, 9, 11, 15, 19, and 21 as well as chromosome 18. No subclones were found in the PB of this MGUS case and none of the BMA altered cells carry the chromosome 8 deletion found in the PB sample (Figure S2). Taken together, these data provide a sample of the genomic landscape of detected MM CTCs and BMPCs with alterations consistent with genomics observed in the previous myeloma single-cell studies using various CTC detection methods and sequencing approaches [3,14,18,19,52,53].

### 3.5. Mapping scCNV Events to FISH Cytogenetics for Clinical Validation

Towards validating the clinical application of the single-cell morphogenomic profiling method, we looked to see whether the same cytogenetic profiles detected by clinical FISH diagnostic assays are found among scCNV profiles of MM CTCs and malignant BMPCs. From clinical diagnostic FISH and karyotyping results (Table 1 and Figure 4A), all the five patients in this cohort were negative for 6/12 (50%) of the common cytogenetic aberrations namely cyclin D1, all three common translocation-based events, FGFR3 gain (via Trisomy 14), and 1q32 loss. MM01 was positive for CCND1 (11q) gain and 13q loss, MM02 was pos positive for gains of 1q21, 11q, 14q, 17p, and 13q loss. MM03 was positive for 17p and 13q losses while MM04 and MGUS were positive for only 11q gain (Figure 4A).

**Figure 5:**
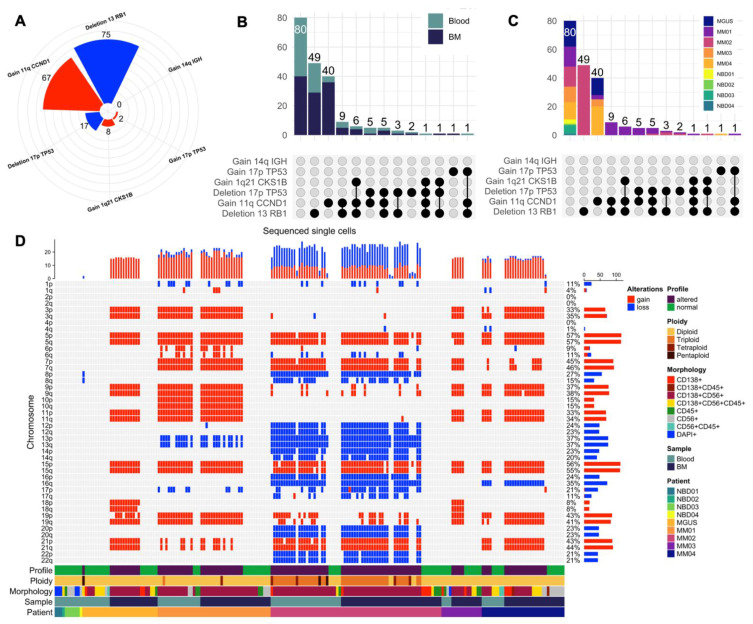
Correlation of scCNV events with diagnostic cytogenetic aberrations for clinical validation. (**A**) Intersect analysis mapping co-occurrence of FISH positive events in scCNV across PB and BMA samples. (**B**) Distribution of positive FISH events in scCNV across patients. (**C**) Enumeration of scCNV harboring indicated FISH cytogenetic aberrations observed across the patient samples. Blue = loss, (**D**) Chromosomal alterations across scCNV profiles of sequenced cells from all patient samples.

To evaluate how well the clinically positive cytogenetic events map to the single-cell profiles detected with the HDSCA-based morphogenomic approach, we quantified the total number of MM CTCs and BMPCs from paired patient samples (Figure 4B) with mapped cytogenetic aberrations (Figure 4A). As presented in (Figure 5A), 75 cells harbor hemizygous loss of 13q (22 in MM01 and 53 in MM01), 67 cells harbor 11q gain (12 in MGUS, 30 in MM02, 1 in MM02, 5 in MM03, 19 in MM04), 17 cells harbor 17p loss (12 in MM01, 5 in MM02), while 1q21 and 17p gains are found in 8 cells (7 in MM01, 1 in MM02) and 2 cells (1 in MM02, 1 in MM04), respectively, with 14q gain being the only common cytogenetic abnormality not found in any of the scCNV profiles (Figure 5A). Concordant with clinical cytogenetics, our CNV analysis found at least one cell carrying the common alterations identified by clinical FISH analysis, except for MM02, where no cells with 14q gain were found. Furthermore, when more than one cell harbors cytogenetic aberrations, they are present in both PB and BMA samples for that patient (Figure 5B) confirming that the detected MM CTC has the same genomic clonality as tumorigenic BMPCs analyzed in diagnostic cytogenetics.

Co-occurrence analysis confirmed that the common cytogenetic events are mapped to the scCNV profiles (Figure 5B,C) and identified additional subclones harboring aberrations dubbed negative in diagnostic cytogenetics (Figure 5D). While clinical cytogenetics found that MM01 is positive for only 11q gain and 13q loss, the patient carries malignant PCs with 17p loss (12 cells), and 1q gain (7 cells). A total of 49 altered cells in MM02 harbor only 13q loss, and one cell in MM04 harbors 17p gain, which is negative in clinical cytogenetics. Loss in 4p (location of FGFR3) was found in two cells from MM04 PB and in one cell in MM01 BMA, suggesting the two patients carry this alteration that clinical FISH and cytogenetic analysis identified as negative.

Together, the scCNV data presented reproducibly map the clinically observed chromosomal alterations to the single-cell CNV genomic events observed in MM CTCs and malignant BMPCs detected with our single-cell assay analysis and further delineate additional subclonal events not detectable by standard diagnostic cytogenetics.

## 4. Discussion

PC malignancies are highly heterogeneous in their genomics and patient risk both in pre-malignant states (MGUS and smoldering MM [SMM]) and in malignant conditions (NDMM and RRMM) thus presenting significant challenges both in early disease detection and therapeutic intervention [52]. Consequently, myeloma remains an incurable disease despite advances in therapeutic modalities as a vast majority of patients eventually relapse due in large part to persistent malignant clones that escape immune surveillance and therapeutic targeting [9,10,11]. Robust delineation of the complex clonal and subclonal heterogeneity in myeloma requires single-cell approaches capable of combined cell morphotype and genomic copy number analysis, providing the opportunity to detect rare cells in an unbiased manner with high sensitivity to identify rare subclones in the patient’s PB for longitudinal disease monitoring of slow-progressing PC neoplasms. Since current clinical standard practice relies on flow cytometry and enrichment-based methods, the identification of malignant cells in myeloma remains a challenge, particularly in the blood of patients with a low population of CD138+ cells that can be isolated and characterized. While flow cytometry is rapid, cheap, and has demonstrated clinical utility in bone marrow samples, its low sensitivity presents analytical limitations for utility in peripheral blood. There remains a significant unmet need for an enrichment-free, highly sensitive, blood-based single-cell method of detecting and characterizing rare myeloma clones.

This study reports the technical and clinical validation of a highly sensitive and specific enrichment-free 4-plex morpho-genomic methodology as a robust approach to detect and characterize circulating rare cells in the PB of patients with PC malignancies. Using paired PB and BMA samples from a cohort of four NDMM patients, one MGUS patient, and four control NBDs and applying HDSCA’s unbiased approach for rare single-cell detection and genomic validation via single-cell sequencing of candidate rare cells, we describe the morphotypes of cells detected using a four-plex immunofluorescence panel comprised of DAPI, CD138, CD56, CD45. We further characterize CD138+ candidate MM CTCs and BMPCs in the PB of NDMM and MGUS patients relying on CD56 expression to morphologically discriminate malignant from normal PCs. Consistent with previous observations, the CD138+ compartment of PCs is heterogeneous in CD138, CD56, and CD45 expression with variable cell morphologies observed between PB and bone marrow [25]. Furthermore, enumeration of the MM CTCs can stratify myeloma patients from NBD using a threshold of >3 CD138+ cells/mL PB. Both MM CTCs and BMPCs in the CD138+ population include subsets of CD138+ only, CD138+CD56+, CD138+CD45+ cells with both small and large morphology (in comparison to common WBCs), and with the canonical characteristic of pericentric nuclei as observed in clinical pathology and confirmed previously [25].

We found candidate PCs in the PB with varying CD138 intensities including CD138− candidate PCs detected based on their large size and eccentric nuclei as compared to surrounding common WBCs, consistent with prior observations [25,36,38,50]. Next-generation single-cell sequencing for CNV profiling provided genomic validation of candidate aberrant cells. Whole-genome sequencing of MM CTC and BMPC candidates revealed aberrated CNV profiles in four NDMM patients and one MGUS case with four NBDs used as controls. In NDMM patients, scCNV genomic analysis validated PC aberrancy in candidate MM CTCs harboring clonality consistent with the paired BMPCs. For clinical validation, we correlated clinical cytogenetic observations to our scCNV data and found concordance of genomic scCNV to clinical cytogenetic results, thus validating the technical capability for identification of rare clones that are informative for clinical practice in PC neoplasms. Notably, the identification of rare clones carrying genomic events not detectable by standard clinical methods is a key strength of this work for future clinical utility in rare cell events contents like MGUS PB and minimal residual disease detection.

There are notable limitations in this study. The small patient cohort size minimizes the extent of biological interpretation and warrants a large-scale study to validate the results presented in this work. Further, the addition of SMM, RRMM, and PCL patient samples would strengthen the observations made here and expand the domain of clinical application for this four-color enrichment-free assay in the ability to detect and describe rare myeloma cells across the progression spectrum. Additionally, the low pass single-cell sequencing conducted here was unable to detect IGH translocations, while future studies will establish the capability to detect translocations as part of the presented morphogenomic profiling, allowing for the direct utility to the hyperdiploid and trisomy myeloma.

Despite the noted limitations, the strengths of this study are in the genomic data that provided both technical and clinical validation for the four-color unbiased immunofluorescence assay built with markers that have been extensively studied and validated as part of clinical histopathology panels [54]. The data presented use patient samples showing direct clinical application. We sampled both bone marrow and PB to demonstrate the robustness in both morphological and genomic characterization of PCs. Beyond NDMM, we show further evidence for use in ultra-rare event samples through the incorporation of MGUS PB and BMA to demonstrate potential in early detection and identified a genomically altered clone. We believe that future work in large cohorts will establish clinical utility for this technology as a blood-based liquid biopsy method to delineate rare genomic subclones in myeloma patients. Additional significant implications include early diagnosis and disease monitoring in contexts where patients are expected to have a low abundance of malignant cells. HDSCA-based morphogenomic analysis provides an alternative method to deconvolute the genomic heterogeneity of myeloma and has the potential to serve as a supportive tool for clinical decisions.

## 5. Conclusions

This study provides technical and initial clinical validation of a slide-based and enrichment-free single-cell immunofluorescence assay for the morphogenomic detection and characterization of normal and aberrant PCs from blood and bone marrow samples of NDMM and MGUS patients. By morphological description, next-generation single-cell sequencing, and correlation of cytogenetics patient data, we demonstrate that our assay recapitulates the detection of common genetic aberrations currently used as the standard for diagnosis and disease monitoring. Concordance analysis further identifies additional rare genetic clones not detected by conventional clinical FISH and karyotyping methodologies. While additional large-scale validation studies are needed to demonstrate the clinical utility of this assay as an approach with the potential to support clinical decisions, particularly in conditions with rare and ultra-rare cells.

## Figures and Tables

**Table 1:** Patient Demographics and Clinical Characteristics.

	MGUS	MM01	MM02	MM03	MM04
Age	78	80	63	54	66
Sex	Male	Male	Female	Male	Male
Diagnosis	MGUS	NDMM	NDMM	NDMM	NDMM
Ig Isotype	IgG*k*	IgG*k*	IgG*k*	IgG*k*	IgA*k*
Percent BMPC in the aspirate	1	14	15	30	8
Percent Aberrant PC from the total PC BM compartment	92	64.5	95.2	98.8	98.2
Flow CD138	Positive	Positive	Positive	Positive	Positive
Flow CD56	Positive	Positive	Positive	Negative	Positive
Flow CD45	Positive	Positive	Negative	Positive (dim)	Negative
M-Spike (g/dL)	0.7	1.6	0.4	2.9	4.3
sFLC ratio	8.14	93.43	186.84	17.28	6.17
Karyotype	Normal	NA	Normal	Hypodiploid	Normal
FISH (Positive)	Three copies of CCND1	Three copies of CCND1; Monosomy 13	Three copies of EGFR3 and CCND1; trisomies 1 and 17; monosomy 13	Monosomies 1, 13, and 17; loss of one copy of IGH	Three copies of CCND1
Clinical Presentation	Low-risk MGUS for progression to MM by PETHEMA [46] criteria	Patient with standard-risk myeloma achieved complete remission after initial therapy with carfilzomib, lenalidomide, dexamethasone	Patient with standard-risk myeloma achieved a partial response after initial therapy with carfilzomib, lenalidomide, dexamethasone	Patient with high-risk myeloma achieved complete remission after therapy with carfilzomib, lenalidomide, dexamethasone but passed away with myeloma progressive disease 21 months after diagnosis	Patient with standard-risk myeloma achieved complete remission after initial therapy with carfilzomib, lenalidomide, dexamethasone

sFLC: serum-free light chain, NDMM: newly diagnosed multiple myeloma, MGUS: monoclonal gammopathy of undetermined significance, PC: plasma cell, BMA: bone marrow aspirate, dim: dim marker expression as defined by flow cytometry.

## Data Availability

All data discussed in this manuscript is included in the main manuscript text and Supplementary Material. The images of the single cells are available through the BloodPAC Data Commons Accession ID “BPDC000123”.

## Supplementary Materials

The following supporting information can be downloaded at: https://www.mdpi.com/article/10.3390/curroncol29050242/s1, Figure S1: Assay validation of markers in spiked NBD samples. A. Representative images at 100× magnification of Jurkat, U266, and MM.1S pure cell lines stained with CD138 (yellow) and DAPI (blue). B. Cell density distribution by CD138 intensity across cell lines; Jurkat (dark red), U266 (green), MM1s (gold). C. Comparative quantification of CD138 intensity in cell lines. D. Representative images of U266 spiked in NBD cells stained with CD138 (red), CD45 (green). All nucleated cells DAPI positive (blue). Figure S2: Heatmap representation of all sequenced cells and associated chromosomal alterations across all patients and sample types.
