# Supplementary material for: Enrichment-Free Single-Cell Detection and Morphogenomic Profiling of Myeloma Patient Samples to Delineate Circulating Rare Plasma Cell Clones
Source: Curr Oncol. 2022 Apr 21;29(5):2954–72. doi: 10.3390/curroncol29050242 (PMC9139906; doi:10.3390/curroncol29050242)
Supplement: Supplementary file 1 [file curroncol-29-00242-s001.zip › curroncol-1633389-supplementary.pdf]

Current Oncology

Article

Enrichment-free Single-cell Detection and Morphogenomic Profiling of Myeloma Patient Samples to Delineate Circulating Rare Plasma Cell Clones

Libere J. Ndacayisaba, Kate E. Rappard, Stephanie N. Shishido, Carmen Ruiz Velasco, Nicholas Matsumoto, Rafael Navarez, Guilin Tang, Pei Lin, Sonia M. Setayesh, Amin Naghdloo, Ching-Ju Hsu, Carlisle Maney, David Symer, Kelly Bethel, Kevin Kelly, Akil Merchant, Robert Orlowski, James Hicks, Jeremy Mason, Elisabeth E. Manasanch and Peter Kuhn

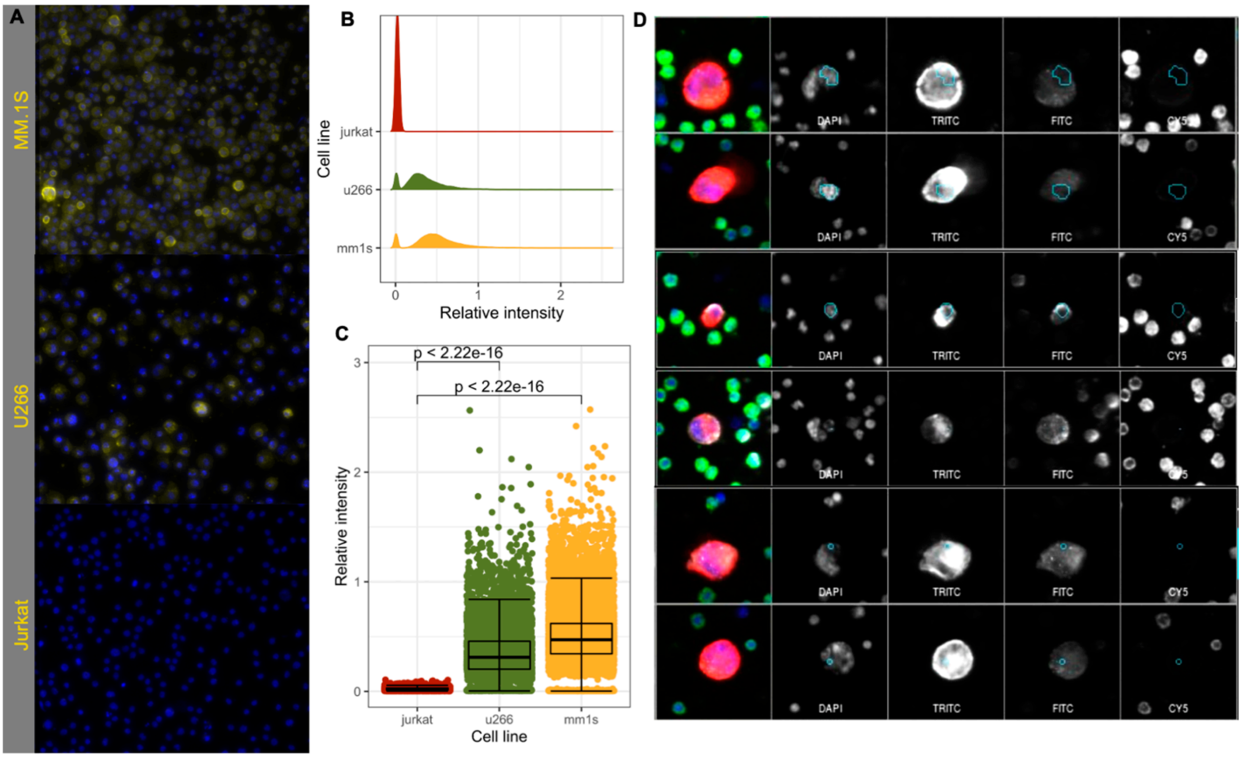

Figure S1. Assay validation of markers in spiked NBD samples. **A**. Representative images at 100x magnification of Jurkat, U266, and MM.1S pure cell lines stained with CD138 (yellow) and DAPI (blue). **B**. Cell density distribution by CD138 intensity across cell cell lines; Jurkat (dark red), U266 (green), MM1s (gold). **C.** Comparative quantification of CD138 intensity in cell lines. **D**. Representative images of U266 spiked in NBD cells stained with CD138 (red), CD45 (green). All nucleated cells DAPI positive (blue).

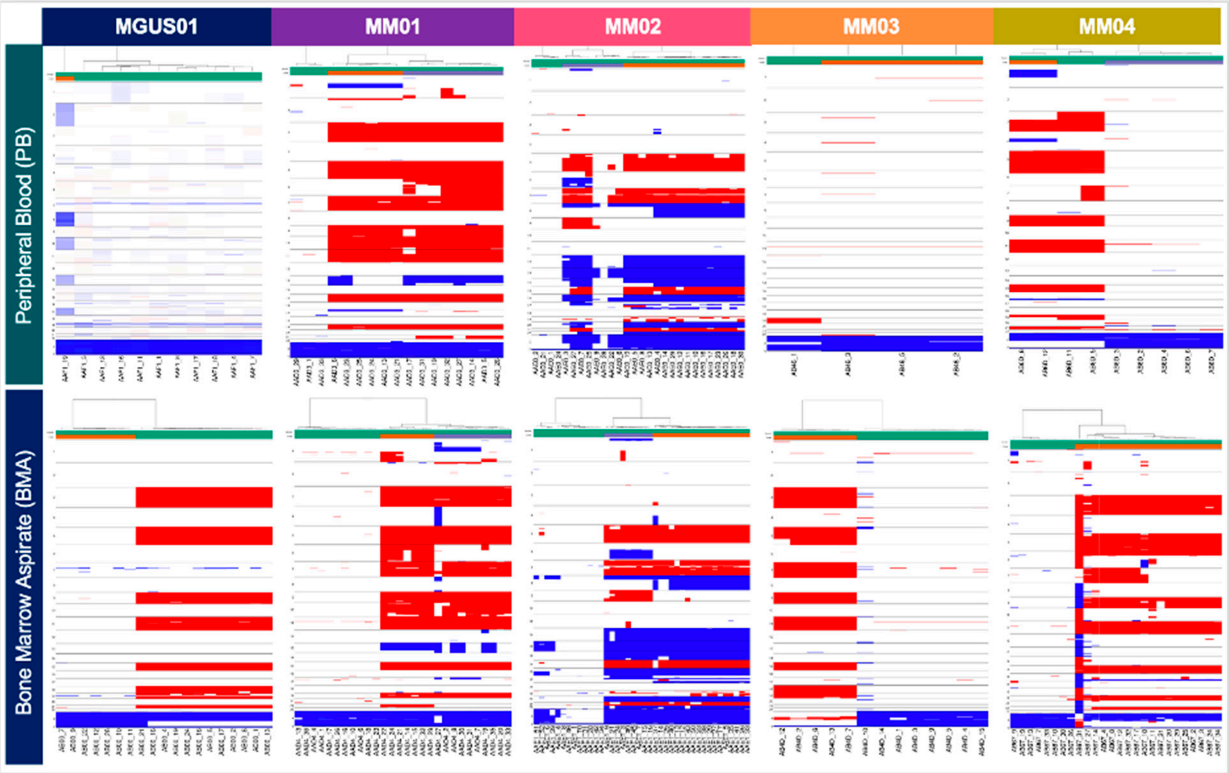

Figure S2. Heatmap representation of all sequenced cells and associated chromosomal alterations across all patients and sample types.
